# Deep learning-based breast region segmentation in raw and processed digital mammograms: generalization across views and vendors

**DOI:** 10.1117/1.JMI.11.1.014001

**Published:** 2023-12-28

**Authors:** Sarah D. Verboom, Marco Caballo, Jim Peters, Jessie Gommers, Daan van den Oever, Mireille J. M. Broeders, Jonas Teuwen, Ioannis Sechopoulos

**Affiliations:** a Radboud University Medical Center, Department of Medical Imaging, Nijmegen, The Netherlands; b Radboud University Medical Center, Department for Health Evidence, Nijmegen, The Netherlands; c Dutch Expert Centre for Screening (LRCB), Nijmegen, The Netherlands; d Netherlands Cancer Institute, Department of Radiation Oncology, Amsterdam, The Netherlands; e Memorial Sloan Kettering Cancer Center, Department of Radiology, New York, New York, United States; f University of Twente, Multi-Modality Medical Imaging, Enschede, The Netherlands

**Keywords:** mammography, segmentation, U-Net, deep convolutional neural network, pectoral muscle

## Abstract

**Purpose:**

We developed a segmentation method suited for both raw (for processing) and processed (for presentation) digital mammograms (DMs) that is designed to generalize across images acquired with systems from different vendors and across the two standard screening views.

**Approach:**

A U-Net was trained to segment mammograms into background, breast, and pectoral muscle. Eight different datasets, including two previously published public sets and six sets of DMs from as many different vendors, were used, totaling 322 screen film mammograms (SFMs) and 4251 DMs (2821 raw/processed pairs and 1430 only processed) from 1077 different women. Three experiments were done: first training on all SFM and processed images, second also including all raw images in training, and finally testing vendor generalization by leaving one dataset out at a time.

**Results:**

The model trained on SFM and processed mammograms achieved a good overall performance regardless of projection and vendor, with a mean (±std. dev.) dice score of 0.96±0.06 for all datasets combined. When raw images were included in training, the mean (±std. dev.) dice score for the raw images was 0.95±0.05 and for the processed images was 0.96±0.04. Testing on a dataset with processed DMs from a vendor that was excluded from training resulted in a difference in mean dice varying between −0.23 to +0.02 from that of the fully trained model.

**Conclusions:**

The proposed segmentation method yields accurate overall segmentation results for both raw and processed mammograms independent of view and vendor. The code and model weights are made available.

## 1 Introduction

Automatic image processing and analysis of digital mammograms (DMs), such as computer-aided detection (CAD), CAD diagnosis, and breast density estimation, have become an important part of breast care.^1^^–^^3^ Most of these methods require a prior segmentation of the mammogram into background, breast, and pectoral muscle areas. The segmentation of the breast is a necessary step to extract image features only from the breast, avoiding the background and, if depicted, the pectoral muscle. If not correctly removed, the pectoral muscle could introduce important biases in any automated system aimed at the analysis of the breast tissue.^1^^,^^4^

Several methods have been proposed to segment mammograms. Most of these methods are designed for digitized screen-film mammograms or raw (for processing) DMs^5^^–^^8^ and mainly focus on only the mediolateral oblique (MLO) view.^5^^,^^7^ Screen-film mammography has widely been replaced by digital mammography, making methods that work solely for digitized screen-film mammography less relevant. Raw (for processing) DMs are often used for physics-based analysis, such as for breast density quantization, but they are not saved in clinical and screening practice, making segmentation methods dedicated to raw mammograms of limited use (especially in large, retrospective studies). Therefore, segmentation methods able to analyze processed (for presentation) DMs, instead of raw data, are needed. However, this shift in data type introduces a new challenge in generalizing across different manufacturers. This is because different vendors use different postprocessing algorithms to convert the raw x-ray projection data to DMs for display, resulting in vendor specific differences in processed images with respect to gray levels, contrast, and texture.^9^ Therefore, a segmentation method that is intended for processed DMs should be designed to handle these differences to generalize well across DMs of different vendors. Finally, although most previously developed methods have focused on only the MLO view, the cranio-caudal (CC) view is also acquired during a standard mammographic examination. In contrast to the MLO view, this view does not always depict the pectoral muscle, and when it is present, it is often small. This makes the automated segmentation of the pectoral muscle in CC images challenging. However, there are cases in which the pectoral muscle is clearly depicted in the CC view and can therefore influence image analysis. For these cases, therefore, it would be beneficial to automatically detect the pectoral muscle also in CC images.

Therefore, considering the limitations in existing methods, the aim of this study is to develop a model to segment the breast region and the pectoral muscle that is applicable to both raw and processed DMs, designed to generalize across models of different vendors and across both CC and MLO views. The code and trained model weights of the model are available at https://github.com/radboud-axti/maseg.

## 2 Methods

A convolutional neural network (CNN) was trained in a supervised fashion to segment mammograms into background, breast, and pectoral muscle areas. For this, eight different datasets were used to train and test the model on mammograms from different vendors. During training, several augmentations were used to increase the model generalization across the contrast differences among vendors. Furthermore, a hyperparameter search was done with two different losses to compensate for the pectoral muscle class imbalance introduced by training a single model for both CC and MLO views. To evaluate the appropriateness of our model, several experiments were performed; these aimed at evaluating the model performance on generalization across views and vendors, the effect of including raw images, and the potential performance of generalization to new vendors.

### 2.1 Datasets

For training, validation, and testing, eight different datasets of mammographic images were used: two consisted of public datasets, mini-MIAS^10^ and INbreast,^11^ and six were datasets of different vendors. An overview of these datasets is shown in Table 1. One to 36 images from each woman are included in the datasets with a median of four images, and in the Hologic, GE, and Siemens datasets, some women had multiple exams acquired on different dates. All datasets consist of 50% MLO images, except the INbreast dataset, which consists of only MLO images. Of the eight datasets, four also include the corresponding raw images. All images were linearly resampled to have a pixel spacing of 400  μm.

**Table 1:** Description of the eight datasets that were used for training, validation, and testing. INbreast and mini-MIAS are publicly available datasets. The mini-MIAS dataset contains only CC images.

	No. images	No. women	DM/SFM	No. raw images	Pectoral muscle visible		Pixel spacing (μm)	Year	Country
					MLO (%)	CC (%)			
Hologic	1012	195	DM	1012	99	36	70 or 65.5	2004 to 2009	The Netherlands
IMS Giotto	821	203	DM	821	99	26	82.9	2022	Italy
Fuji	756	200	DM	No	99	18	50	2022	Germany
GE	720	104	DM	720	98	41	100 or 94	2000 to 2019	The Netherlands
INbreast^11^ (Siemens)	410	108	DM	No	99	8	70	2008 to 2010	Portugal
Mini-MIAS^10^	322	161	SFM	No	98	—	200	1994	United Kingdom
Siemens	268	40	DM	268	100	40	85	2001 to 2019	The Netherlands
PlanMed	264	66	DM	No	100	21	83	2018 to 2021	Finland
Total	4573	1077		2821	99	28			

DM, digital mammography; SFM, screen film mammography; MLO, medio-lateral oblique; and CC, cranio-caudal.

#### 2.1.1 Ground truth

**Fig. 1:**
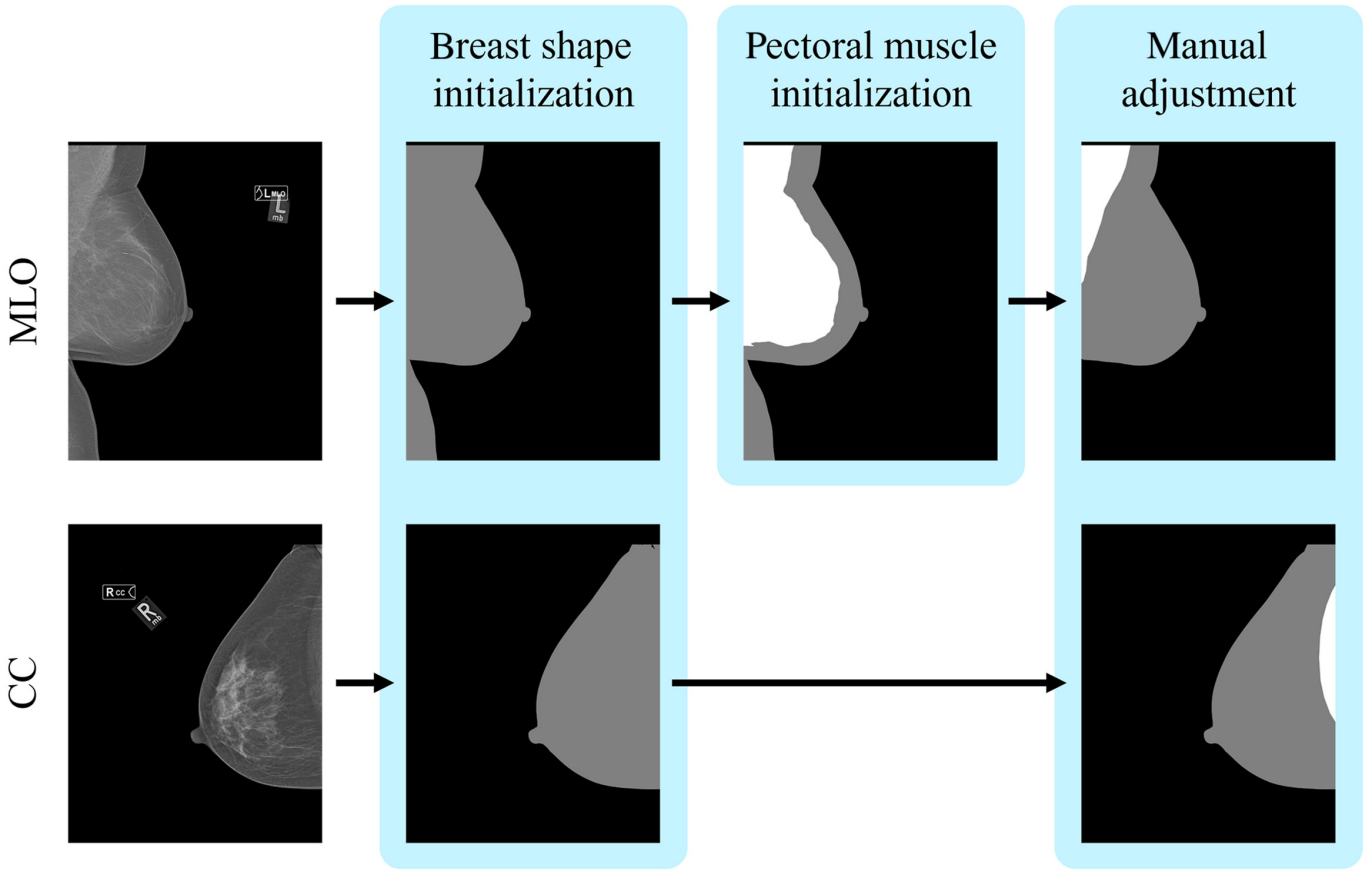
Annotation process for processed images with one or two initialization steps and manual adjustment.

A segmentation mask with pixel-wise ground truth for background, breast, and pectoral muscle (when present) was made using the processed mammograms and screen film mammograms (SFMs). If present, the labels were then applied to the corresponding raw images because no positional shifts are performed by any of the image processing algorithms. Segmentation masks were created in two or three steps: first, initialization of the breast boundary by Otsu thresholding;^12^ second, a pectoral muscle initialization with Otsu thresholding only for MLO images; and finally, a manual adjustment of the mask, as shown in Fig. 1. The pectoral muscle initialization in MLO images was done by reapplying the Otsu thresholding method after excluding the background for seven of the eight datasets. For the MLO view of the INbreast dataset,^11^ instead, the already publicly available annotations of the pectoral muscle were used as the initialization. Finally, each segmentation mask was checked visually and adjusted manually using ITK-SNAP 3.6.0^13^ by one of four medical imaging scientists with experience in mammography.

#### 2.1.2 Data split

Each dataset was split randomly on a patient level into at least 100 test images, 80 validation images, and the rest in training. In total, this led to a training set of 3116 images, a validation set of 645 images, and a test set of 812 images, as shown in Table 2. The same split was used for all experiments.

**Table 2:** Split of each of the eight datasets into a training, validation, and test sets.

	Train		Validation		Test	
	No. women	No. images	No. women	No. images	No. women	No. images
Hologic	155	832	18	80	22	100
IMS Giotto	158	638	20	81	25	102
Fuji	152	576	23	80	25	100
GE	75	540	17	80	12	100
INbreast	62	224	22	80	24	106
mini-MIAS	71	142	40	80	50	100
Siemens	12	80	13	84	15	104
PlanMed	21	84	20	80	25	100
Total	706	3116	173	645	198	812

### 2.2 Network

#### 2.2.1 Architecture

The multiclass segmentation was performed using a U-Net^14^ with four downsampling blocks of two convolutional layers each with a kernel size of three, and a max-pooling layer with a kernel size of two and stride of two. The initial number of convolutional filters was set to 64 and doubled at each scale. The four upsampling steps consisted of a bilinear upsampling, a convolutional layer with a kernel size of unity to reduce the number of filters by a factor of two, and two convolutional layers with a kernel size of three. Before the final SoftMax layer, a convolutional layer (three filters, unity kernel size) was included. Dropout regularization (probability of 0.5) was applied in this layer to allow for an improved generalization and to improve the training optimization of the deeper layers. All activation functions were rectified linear units, and weights were initialized with Kaiming uniform initialization.

#### 2.2.2 Augmentations

**Fig. 2:**
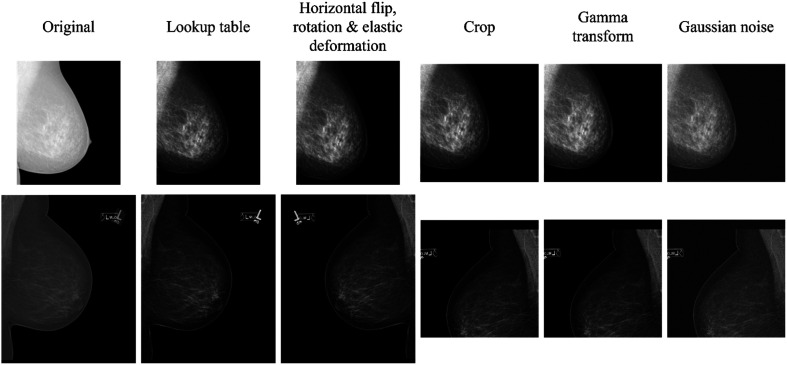
Examples of two training images after all data augmentation steps. The examples show the variety in gray level intensity and contrast between the images.

Processed DMs of different vendors have different intensity and contrast properties mainly due to differences in processing of the raw images. To generalize over these different properties, several types of data augmentation were used as a regularizer during training, as shown in Table 3. For each training image, a random look-up table was chosen from the choices provided in its DICOM header, with a jitter varying the window center and window width slightly. This was followed by a random horizontal flip, a random rotation, and an elastic deformation.^15^ After these augmentations, a random cropping of the image of 512×512  pixels was performed. The cropping was allowed to go at most 64 (12.5%) pixels outside of the original image boundary and, where necessary, the images was padded with zeros. Finally, a random gamma transform was applied, and Gaussian noise was added. Two examples of the result of these augmentations on mammograms are shown in Fig. 2.

**Table 3:** Augmentations that were performed during training with their corresponding parameter ranges.

Augmentation	Parameters
Random lookup table	±5% of window center and window width
Random horizontal flip	Probability = 0.5
Random rotation	−5 deg to +5 deg
Elastic deformation^15^	α=1, σ=100 and $\alpha_{\text{affine}}=20$
Random cropping	512 × 512 pixels
Random gamma transform	γ∈(0.5,1.5)
Random Gaussian noise	Standard deviation 5% of image range

#### 2.2.3 Losses

If present in the CC view, the pectoral muscle region is often small, comprising a very small area of the overall image and of the breast. To account for this class imbalance, two different weighted losses were used to train the network: weighted cross entropy and weighted focal loss.

The weighted cross-entropy loss is defined as

$$\text{cross-entropy loss}\;(p_{t})=-\overset{3}{\underset{c=1}{\sum }}\alpha_{c}\;\log\;p_{t,c},$$ (1)

$$p_{t,c}=\{\begin{array}{ll} p_{c}, & y=c\\ 1-p_{c}, & \text{otherwise} \end{array},$$ (2)

where $p_{c}$ denotes the probability of a pixel belonging to class c and y is the ground truth of that pixel. Hyperparameters $\alpha_{c}$ weigh the classes, giving a higher or lower weight to a certain class c.

The focal loss^16^ is a variation of the weighted cross-entropy loss, in which high confidence predictions are weighted lower than low confidence predictions by tuning a hyperparameter (γ). The focal loss is defined as

$$\text{focal loss}(p_{t})=-\overset{3}{\underset{c=1}{\sum }}\alpha_{c}{\left(1-p_{t,c}\right)}^{\gamma }\;\log\;p_{t,c},$$ (3)

with hyperparameters $\alpha_{c}$ and γ (γ=1 corresponds to cross-entropy loss). In this study, we used γ=2, as was suggested in the original paper.^14^

The model was trained with the entire training set with both losses and with $\alpha =[\begin{array}{ccc} 1 & 1 & \alpha_{\text{pectoral}} \end{array}]$ such that $\alpha_{\text{pectoral}}\in \{1,1.5,2,2.5\}$. Training was stopped when the validation loss did not improve for 10 epochs. The final loss and $\alpha_{\text{pectoral}}$ were chosen based on the highest segmentation dice coefficient on the entire validation set for the pectoral class and were not modified further when assessing the model performance on the test set.

### 2.3 Experiments

#### 2.3.1 Comparing views and vendors

The model was trained with all eight training datasets including only the processed (and digitized screen film) images, totaling 3116 images using the selected loss and hyperparameters. The segmentation performance of the network was tested on the processed test images of all eight datasets, with the look-up table provided in the DICOM header as the “normal” setting, with no further test augmentation. The performance was quantified by the dice coefficient for each class c present in the image, defined as

DICE(c)=2·|y^c∩yc||y^c|+|yc|, (4)

which compares the class segmentation estimate y^c to the class ground truth $y_{c}$. An overall dice coefficient was computed for each image by averaging the dice coefficients for the breast and pectoral areas weighted by the number of pixels of the corresponding class in the ground truth.

Dice coefficients for CC and MLO view test set images were compared with an unpaired t-test. The class and average dice coefficients of the eight datasets were compared using a one-way ANOVA test and Tukey’s method as a *post hoc* test. All tests were performed with SciPy, and a significance level of 0.05 was used.

As discussed, not all mammograms depict the pectoral muscle. This could lead to the model segmenting the pectoral muscle when it is not present, a false positive, or not segmenting a pectoral muscle when it is present, a false negative. Therefore, in addition to the dice coefficient, the performance in pectoral muscle segmentation was also quantified in terms of the number of false positive and false negative cases. A false positive was defined as an output in which some pixels are classified as pectoral muscle when there is no pectoral muscle present in the ground truth. A false negative was defined as an output in which no pectoral muscle is segmented when there is a pectoral muscle present in the ground truth.

#### 2.3.2 Including raw images in training

In the first experiment, the model was trained only on processed and SFM images. To expand the applicability of the model to raw images also, these were included during training in the second experiment. For this, the model was retrained on the same training set, but additionally including all 2090 available raw images of the training set. The dice coefficients of the 812 processed and 406 raw test images for both models (processed-only training and processed + raw training) were compared with a paired t-test with a significance level of 0.05.

#### 2.3.3 Test for vendor generalization

A third experiment was performed to assess the potential ability of the model to generalize to images from a possible new, unseen vendor or images processed with a new vendor processing algorithm. For this, eight different models were trained, each time including all processed-image-only training sets except for one, the excluded dataset. The resulting model was then tested on the training and test sets of the excluded dataset. For each model, the resulting dice coefficients were compared with the dice coefficients of the test set of the datasets that were used for training with an unpaired t-test. The significance level of 0.05 was adjusted for multiple comparisons with a Bonferroni correction to 0.05/8.

#### 2.3.4 Comparison with Volpara commercial segmentation algorithm

The research version of a commercially available breast density quantification software (Volpara version 3.4, Volpara Health, Wellington, New Zealand) was used to generate a segmentation of all raw images. Volpara returns a segmentation with four classes: background, pectoral muscle, breast tissue with a constant thickness, and breast tissue with a varying thickness. To compare these segmentations to the ground truth masks, both breast tissue classes were combined into one breast tissue class. The resulting dice coefficients were compared with the performance of the model that was trained on both processed and raw images applied to the raw images and corresponding processed images with a dependent t-test.

## 3 Results

The cross-entropy loss with $\alpha_{\text{pectoral}}=2.0$ resulted in the highest pectoral and overall dice coefficient in the validation set (as shown in Table 4) and was thus used for all following models. Higher values of $\alpha_{\text{pectoral}}$ resulted in over-segmentation of the pectoral muscle, whereas lower values resulted in under-segmentation. All following models were trained with a cross-entropy loss with $\alpha_{\text{pectoral}}=2.0$.

**Table 4:** Dice coefficients for the two losses with different values of $\alpha_{\text{pectoral}}$ for the validation set. The pectoral muscle dice was calculated only for the 445/645 images in which the pectoral muscle was visible. Results are given in mean ± standard deviation.

Loss	$\alpha_{\text{pectoral}}$	Breast	Pectoral
Cross-entropy loss	1.0	0.94 ± 0.07	0.56 ± 0.32
	1.5	0.93 ± 0.05	0.58 ± 0.27
	2.0^a^	0.93 ± 0.05	0.63 ± 0.26
	2.5	0.94 ± 0.06	0.60 ± 0.29
Focal loss	1.0	0.92 ± 0.08	0.23 ± 0.29
	1.5	0.96 ± 0.05	0.60 ± 0.34
	2.0	0.93 ± 0.10	0.59 ± 0.31
	2.5	0.93 ± 0.07	0.45 ± 0.26

a Selected hyperparameter for training.

### 3.1 Comparison of Views and Vendors

**Fig. 3:**
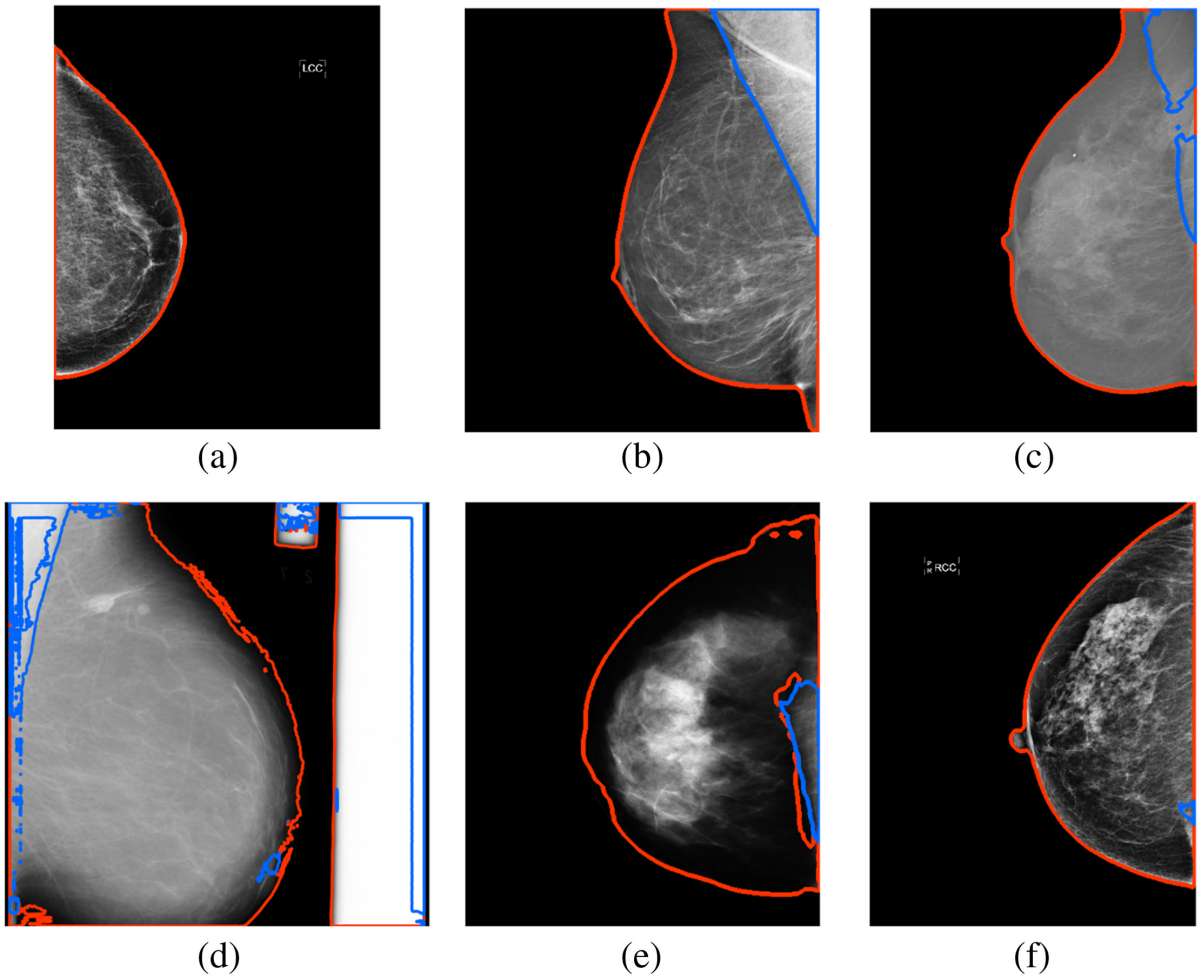
Example of segmentation outputs (a) of a CC view with a high overall dice (0.98) from GE, (b) MLO view with a high overall dice (0.99) from Hologic, (c) MLO view with a median overall dice (0.95) and split pectoral segmentation from Inbreast, (d) MLO view with a low overall dice (0.80) from mini-MIAS CC view with a low overall dice (0.94) from GE, and (f) CC view with a median overall dice (0.99) but false positive pectoral muscle from Hologic. The red line indicates the background-breast edge, and the blue line indicates the breast-pectoral muscle edge.

Table 5 shows the overall and view-specific results from the first experiment. Figures 3(a) and 3(b) show representative examples of segmentation outputs for both the MLO and CC views, respectively. Figure 3(d) shows an example from the mini-MIAS dataset in which the model mistakes the white edges for breast tissue and pectoral muscle, resulting in a low dice coefficient. Figure 3(e) shows a CC example with a low dice coefficient.

**Table 5:** Dice coefficients on the test set for different views. Results are given in mean ± standard deviation. The number of images that contain the pectoral muscle class in the ground truth is given.

View	n	Breast	Pectoral (n)	Overall
MLO	456	0.96 ± 0.04	0.83 ± 0.20 (451)	0.94 ± 0.07
CC	356	0.98 ± 0.02	0.50 ± 0.35 (93)	0.98 ± 0.02
All	812	0.97 ± 0.03	0.78 ± 0.26 (544)	0.96 ± 0.06

MLO, medio-lateral oblique and CC, cranio-caudal.

The overall dice coefficient differed significantly between MLO (0.94±0.07) and CC (0.98±0.02) views, p<0.001, as shown in Table 5. The segmentation of the breast class yielded higher dice coefficients for CC compared with MLO views (p<0.001), and the pectoral muscle dice coefficient was lower for CC than for MLO views (p<0.001).

When the pectoral muscle was present, it was missed in 25/93 (26.9%) CC images, as shown in Table 6. When the pectoral muscle was not missed, the dice of the pectoral muscle in the CC views was 0.68±0.21 (n=68). The pectoral muscle was never missed in MLO images. False positive pectoral muscle segmentation larger than 1% of the breast area occurred in 28/263 (10.6%) of CC images without a pectoral muscle.

**Table 6:** False negative and false positive rates for the pectoral muscle in different views. The size of the pectoral muscle is given in mean percentage of the breast area ± standard deviation.

View	n	Pectoral present	Pectoral size	False negatives	False positives
MLO	456	98.9% (451/456)	21.1% ± 13.6%	0.0% (0/451)	100.0% (5/5)
CC	356	26.1% (93/356)	4.1% ± 3.2%	26.9% (25/93)	44.5% (117/263)

MLO, medio-lateral oblique and CC, cranio-caudal.

The mean overall dice coefficient of the different datasets differed significantly (p<0.001) and ranged from 0.86 to 0.98, as shown in Table 7. The *post hoc* analysis showed that mini-MIAS was significantly different from all others (p<0.001) with the lowest overall dice coefficient (mean ± std. dev) of 0.86±0.09. When only including DMs, the overall dice coefficient (mean ± std. dev) was 0.97±0.03. Furthermore, the *post hoc* analysis also showed that the overall dice coefficients of Siemens and INbreast were statistically different (p=0.017).

**Table 7:** Dice coefficients of the test set for each of the eight datasets, the total test set, and all DMs from the test set, which include all datasets excluding mini-MIAS. Results are given in mean ± standard deviation. The pectoral muscle dice was calculated only for images in which the pectoral muscle was visible. The number of images that contain the pectoral muscle class in the ground truth is given in (n).

Dataset	n	Breast	Pectoral (n)	Overall
Hologic	100	0.98 ± 0.01	0.81 ± 0.30 (68)	0.98 ± 0.02
IMS Giotto	102	0.98 ± 0.02	0.79 ± 0.31 (64)	0.97 ± 0.04
Fuji	100	0.98 ± 0.02	0.87 ± 0.17 (58)	0.98 ± 0.03
GE	100	0.97 ± 0.02	0.81 ± 0.29 (65)	0.97 ± 0.03
INbreast	106	0.99 ± 0.01	0.81 ± 0.24 (57)	0.98 ± 0.02
Mini-MIAS	100	0.91 ± 0.05	0.55 ± 0.15 (97)	0.86 ± 0.09
Siemens	104	0.97 ± 0.02	0.83 ± 0.22 (76)	0.96 ± 0.03
PlanMed	100	0.97 ± 0.02	0.87 ± 0.21 (59)	0.97 ± 0.05
All	812	0.97 ± 0.03	0.78 ± 0.26 (544)	0.96 ± 0.06
All DM	712	0.98 ± 0.02	0.83 ± 0.26 (447)	0.97 ± 0.03

DM, digital mammography.

### 3.2 Including Raw Images in Training

When the raw images were included in training, the overall segmentation dice for raw images improved, from an overall dice of 0.68±0.25 (mean ± std. dev.) with the processed-only-training model to an overall dice of 0.95±0.05 (mean ± std. dev.) (p<0.001), as shown in Table 8. As can also be seen, adding the raw images in training had no effect on the overall performance of the segmentation algorithm on the processed images (p=0.11).

**Table 8:** Dice coefficients of the test set for processed and raw images of each of the eight datasets, the total test set, and the four datasets that have raw images. Results are given in mean ± standard deviation and with the number of images (n).

Dataset	n	Trained with processed only		Trained with processed and raw	
		Processed	Raw	Processed	Raw
Hologic	100	0.98 ± 0.02	0.46 ± 0.14	0.97 ± 0.03	0.95 ± 0.04
IMS Giotto	102	0.97 ± 0.04	0.59 ± 0.27	0.96 ± 0.05	0.95 ± 0.07
Fuji	100	0.98 ± 0.03		0.97 ± 0.03	
GE	100	0.97 ± 0.03	0.90 ± 0.08	0.96 ± 0.04	0.96 ± 0.03
INbreast	106	0.98 ± 0.02		0.96 ± 0.04	
Mini-MIAS	100	0.86 ± 0.09		0.90 ± 0.08	
Siemens	104	0.96 ± 0.03	0.76 ± 0.21	0.96 ± 0.05	0.96 ± 0.04
PlanMed	100	0.97 ± 0.05		0.96 ± 0.06	
All	812	0.96 ± 0.06		0.95 ± 0.05	
Datasets containing processed and raw	406	0.97 ± 0.03	0.68 ± 0.25	0.96 ± 0.04	0.95 ± 0.05

### 3.3 Vendor Generalization

Of the eight models that were trained, three performed significantly better on the excluded dataset than on the included datasets, two had no significant difference, and three performed significantly worse on the excluded set, as shown in Table 9. The differences in mean overall dice were small, varying between −0.23 to +0.02. The largest decrease in performance was found for the model that had to segment the mini-MIAS dataset without having seen it during training.

**Table 9:** Dice coefficients when the model is trained on all processed training images of all datasets, excluding one dataset, and tested on (1) the complete test set excluding the same dataset and (2) the training and test set of the excluded dataset. Results are given in mean ± standard deviation. A Bonferroni corrected significance level of 0.05/8 = 0.00625.

Training		Test set included datasets		Train and Test set excluded dataset		Difference	p
Excluded dataset	n	Dice	n	Dice	n		
Hologic	2284	0.95 ± 0.07	712	0.95 ± 0.03	932	+0.00	0.202
IMS Giotto	2478	0.95 ± 0.07	710	0.88 ± 0.15	740	−0.07^a^	<0.001
Fuji	2540	0.94 ± 0.07	712	0.97 ± 0.03	676	+0.02^a^	<0.001
GE	2576	0.94 ± 0.07	712	0.89 ± 0.10	640	−0.04^a^	<0.001
INbreast	2892	0.95 ± 0.06	706	0.95 ± 0.07	330	+0.00	0.701
Mini-MIAS	2,974	0.97 ± 0.05	712	0.74 ± 0.13	242	−0.23^a^	<0.001
Siemens	3036	0.96 ± 0.05	708	0.97 ± 0.04	184	+0.01^a^	<0.001
PlanMed	3032	0.94 ± 0.07	712	0.97 ± 0.04	184	+0.02^a^	<0.001

a Statistically significant difference.

### 3.4 Comparing with Volpara Segmentation

The mean overall dice coefficient of Volpara applied to the raw images was 0.98±0.03, which was higher than the overall dice coefficient of the model trained with both processed and raw images for the corresponding processed images (0.96±0.04) and raw images (0.95±0.05, both p<0.001).

## 4 Discussion

The aim of this study was to propose a segmentation method suited for both raw and processed DMs that can generalize across CC and MLO views and across different vendors. The model provided a good overall performance regardless of raw/processed image, view, and vendor, achieving an overall dice of >0.95 on average across all of these conditions. When excluding the mini-MIAS dataset with SFMs and evaluating on only processed DMs, the mean overall dice surpassed 0.97.

Although the segmentation performance is satisfactory for both views, there was a difference between segmenting CC and MLO views, especially in the segmentation of the pectoral muscle. The performance of pectoral muscle segmentation was lower and more widely distributed in CC images compared with that of MLO images. There are three main possible explanations for this. First, the pectoral muscle was not always present in the CC views, being in only 28% of the images compared with 99% of the MLO images. Therefore, the model was trained on a much larger dataset when segmenting pectoral muscles in MLO images compared with CC images. Second, the area of the pectoral muscle, with respect to that of the breast region, was much smaller in CC views (4.7%±3.0%) compared with MLO views (21.4%±13.5%). As seen in Eq. (4), a smaller area in the annotation can make the dice coefficient more sensitive to small errors in segmentation. Third, 26.9% of the CC views with an annotated pectoral muscle were false negatives. These images have a pectoral dice of 0, greatly decreasing the mean pectoral dice. If the pectoral muscle is found in a CC view image, it is segmented with a lower, but satisfactory, dice score compared with the MLO view. Of course, given the small size of the pectoral muscle in the CC view, the impact of a subpar pectoral segmentation on misclassified number of pixels and on the overall dice is minor.

The mean overall dice coefficients across the different DM datasets varied from 0.86 to 0.98. These differences are most likely not clinically relevant. An exception to this is the mini-MIAS dataset. With 0.86, the mean overall dice for this dataset was considerably lower than that for the other datasets. This relatively low performance can be explained by the underrepresentation of SFMs in the training set. The public dataset mini-MIAS is the only included dataset that consists of digitized SFMs instead of DMs.

In the second experiment, raw images were included in training to expand the applicability of the segmentation model from only processed DMs to processed and raw DMs. In clinical practice, raw images are often not saved. However, some physics-based analysis, such as breast density quantization, is performed in real-time on raw images, whereas in research, raw images can play an important role due to their pixel values representing actual attenuation information of different tissues. Without including raw images in training, the performance of the segmentation on raw images was much lower than for processed images and was not sufficient. Including raw images during training, instead, increased the performance on raw images and almost closed the gap in performance between processed and raw images. Moreover, including raw DMs in training also did not significantly decrease the performance on processed DMs. In short, this experiment shows that the model trained on processed and raw mammograms can be applied to both processed and raw DMs without the need to specify the type of image.

The final experiment illustrates how the model might perform when presented with mammograms from a different, unseen, vendor. When the mini-MIAS dataset was excluded, the model was trained only on DMs and, therefore, did not generalize well to the SFMs in the mini-MIAS dataset. This replicates what was also found during the first experiment. Figure 3 shows the clear differences of the SFMs in the mini-MIAS dataset (d) to the DMs in the other datasets. The SFMs in the mini-MIAS dataset have a dark breast edge and include bright labels and edges of the screen film. However, for the other seven datasets, all containing DMs, the difference in mean overall dice between included and excluded datasets varied between −0.07 and +0.02. Excluding IMS Giotto data seems to lead to the poorest performance of the DMs sets for the excluded set. Overall, this experiment shows that the model is likely to generalize well to DMs of other vendors or of new image processing algorithms from the vendors included in this study.

Comparing the segmentation results with those by Volpara showed a minor difference in performance. In contrast to the proposed segmentation method, however, Volpara runs on only raw images. There are several other studies that proposed a method for segmenting mammograms, based on rule-based and machine learning approaches. A rule-based approach by Pawar et al.^17^ used an adaptive threshold for segmenting the background and a region growing method with an automatically selected starting point for pectoral muscle segmentation. The method was tested on only scanned MLO SFMs and had a dice of 0.955 for pectoral muscle segmentation. This is higher than that found in this study for pectoral muscle segmentation. The difference can be explained by the heterogeneity of the images in this study as CC images and MLO images without a pectoral muscle were also included. Zebari et al.^5^ introduced a segmentation method for both scanned MLO screen-film mammograms and digital MLO mammograms. The background segmentation was also done with an adaptive threshold, and local feature extraction and a two-layer neural network were used to segment the pectoral muscle. The average dice coefficient for breast segmentation with this method was 0.991, which is in the range of the dice coefficient of breast segmentation of this study.

Other studies using a CNN to segment mammograms found lower or comparable segmentation performance. Two articles both used a U-Net with a ResNet encoder; one designed for raw DM MLO images^18^ and the other for both scanned MLO film mammograms and digital MLO mammograms^19^ had an overall dice coefficient of 0.949±0.019 and 0.92, respectively, which is lower than the overall dice found in the current study. Rampun et al.^7^ introduced a CNN inspired by holistically nested edge detection for finding the pectoral boundary in both scanned MLO film mammograms and digital MLO mammograms. The pectoral segmentation had a dice coefficient of 0.975±0.063, which is higher than the average dice coefficients for MLO images in our study of 0.835±0.196. Note that Rampun et al.^7^ corrected the output by selecting only the pectoral muscle segmentation that had the longest edge. The method proposed in this study has no such correction. It would be expected that a correction of selecting only the largest connected area could increase the segmentation performance. Overall, the segmentation results of this study fall within the performance range of published segmentation methods and, in contrast to existing methods, can be applied to both raw and processed DMs of different vendors and to both MLO and CC views.

Our study has some limitations, mainly related to the datasets used. One of the limitations of this study is the limited information on the imaged women. All eight datasets were acquired in Europe, and their population varies from screening to diagnostic. Furthermore, every image has only one annotation made by one of four researchers. Therefore, there is no information on the interreader variability to compare the model segmentation performance to. Testing on raw images was also limited because raw images were also available for only four datasets (Hologic, IMS Giotto, GE, and Siemens). Therefore, the performance on raw images could be evaluated only on these four datasets. However, the results of this study suggest good generalization across processed images of different vendors; therefore, it is likely that the model will also generalize well across raw images of different vendors. Finally, the segmentations were evaluated only by the dice coefficients. Although this is an objective measure of segmentation performance, it is not a guarantee for applicability for all downstream tasks.

## 5 Conclusion

The proposed deep learning-based mammogram segmentation method yielded accurate overall segmentation results for raw and processed mammograms for both standard views and was able to generalize well over mammograms from different vendors. The model should perform well on processed DMs of vendors that were not included in this study, whereas its performance on unseen digitized screen-film mammograms might be more limited.

## Data Availability

The code and trained weights for the model trained with processed and raw images (experiment 2) is available at https://github.com/radboud-axti/maseg. Manually annotated masks of the publicly available datasets INbreast and mini-MIAS are available at https://zenodo.org/records/10171732 and https://zenodo.org/records/10149914.
